# Structure and fabrication details of an integrated modularized microfluidic system

**DOI:** 10.1016/j.dib.2015.09.036

**Published:** 2015-10-08

**Authors:** Qingchang Tian, Ying Mu, Yanan Xu, Qi Song, Bingwen Yu, Congcong Ma, Wei Jin, Qinhan Jin

**Affiliations:** a Research Center for Analytical Instrumentation, Institute of Cyber Systems and Control, State Key Laboratory of Industrial Control Technology, Zhejiang University, Hangzhou 310058, Zhejiang, PR China; b College of Life Sciences, Zhejiang University, Hangzhou 310058, Zhejiang, PR China

## Abstract

This article contains schemes, original experimental data and figures for an integrated modularized microfluidic system described in “An integrated microfluidic system for bovine DNA purification and digital PCR detection [1]”. In this data article, we described the structure and fabrication of the integrated modularized microfluidic system. This microfluidic system was applied to isolate DNA from ovine tissue lysate and detect the bovine DNA with digital PCR (dPCR). The DNA extraction efficiency of the microdevice was compared with the efficiency of benchtop protocol.

**Specifications Table**

**Table:** 

Subject area	*Biology*
More specific subject area	*microfluidic*
Type of data	*Table,figure*
How data was acquired	Amplification plots and standard curve plots of qPCR. Digital PCR fluorescent imagines.
Data format	*Raw, analyzed.*
Experimental factors	Use of bovine tissue lysate and ovine tissue lysate to make up different weight ratio (WR) (w/w).
Experimental features	Tissue lysate was introduced into microdevice for DNA isolation and different WR tissue lysates were detected by digital PCR.
Data source location	*Hangzhou, Zhejiang, China.*
Data accessibility	*Data in public repository.*

**Value of the data**

• The structure and fabrication details of an integrated modularized microfluidic system are given.
• Amplification plots and DNA yields resulting from on-chip isolation can be compared with the benchtop procedure.
• Data from qPCR were compared with data from dPCR.

## 1 Data, experimental design, materials and methods

### 1.1 Microdevice design and fabrication

Integration is increasingly recognized as an important technical challenge in lab-on-a-chip device. More efficient and lower cost integrated microfluidic systems decrease or eliminate reliance on traditional lab equipment. Chin et al. [2] integrated new procedures for manufacturing, fluid handling and signal detection in microdevice into a single ‘mChip’ assay to replicate all steps of ELISA. Easley et al. [3] developed an integrated microfluidic genetic analysis system that could extract and purify DNA from crude whole blood sample, carry out PCR-based amplification, following by separation and detection in a manner that allows for microliter samples to be screened for infectious pathogens with sample-in–answer-out results.

**Fig. 1:**
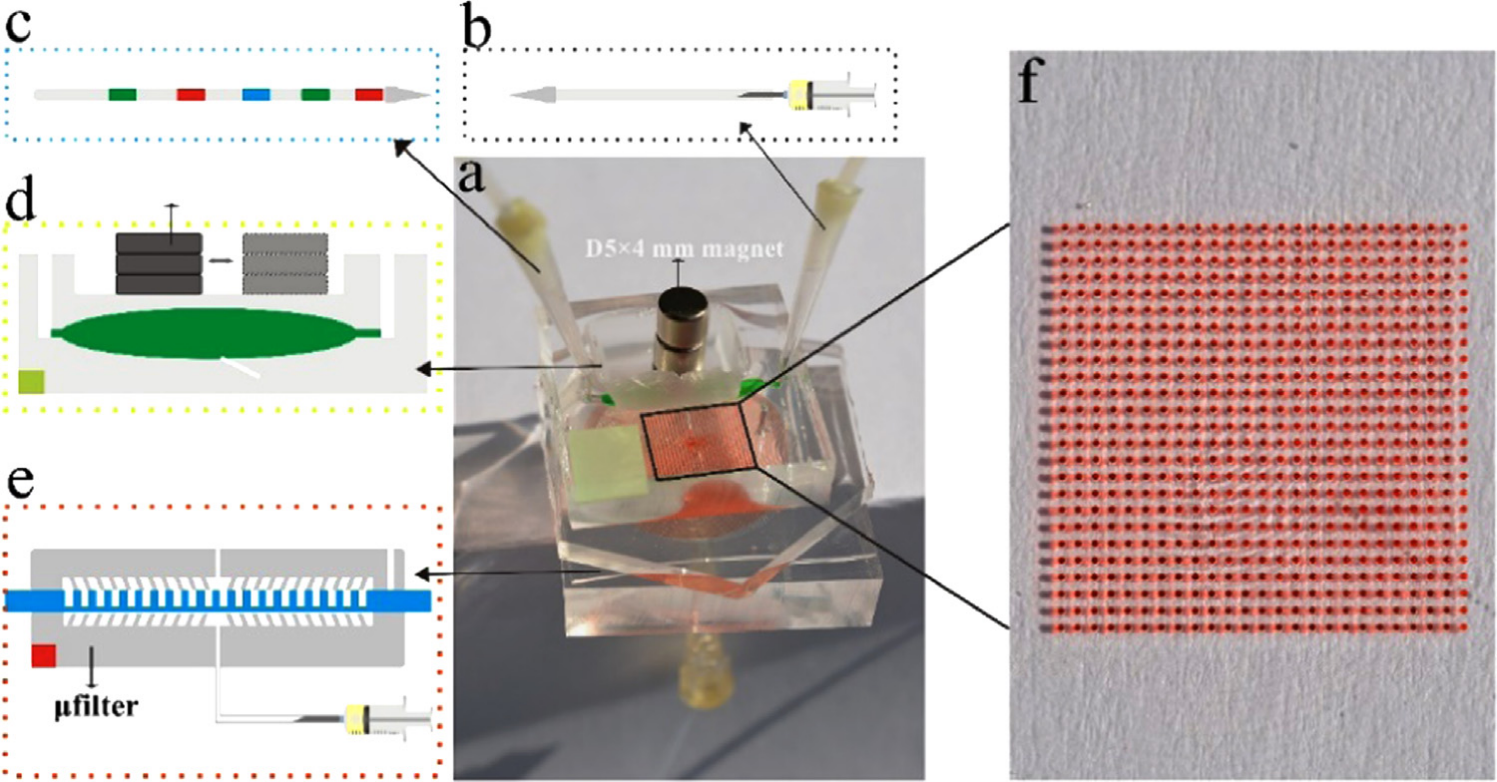
The integrated NA analysis system. (a) The integrated NA analysis system has two distinct functional modules, NA extraction module (green) and dPCR module (red). (b) A Teflon tube connected a syringe to provide negative pressure. (c) A Teflon tube with different reagents (washing buffer and sample lysate) for NA extraction. (d) The NA extraction module with a magnet sliding in a groove. (e) The dPCR module contained a dPCR region and a μfilter with annular duct (tilted "posts" on the μfilter demonstrated the annular duct). (f) The dPCR region with 650 reaction chambers in a square area 15.0 mm×15.0 mm.

We have integrated a NA extraction module and a dPCR reaction module on this chip. As shown in Fig. 1, three washing buffer and sample lysate were preloaded in the Teflon tube one by one with air spacers (Fig. 1c). The NA capture zone was a teardrop-shaped three-way tube (inlet, outlet 1 and outlet 2) (Fig. 1d) with a sizeable magnet. The dPCR module (Fig. 1e) had a dPCR region with 650 reaction chambers (200 μm diameter and 230 μm high) in a square area (15.0 mm×15.0 mm) (Fig. 1f) and a μfilter with annular duct (200 µm in width, 40 µm in depth).

**Fig. 2:**
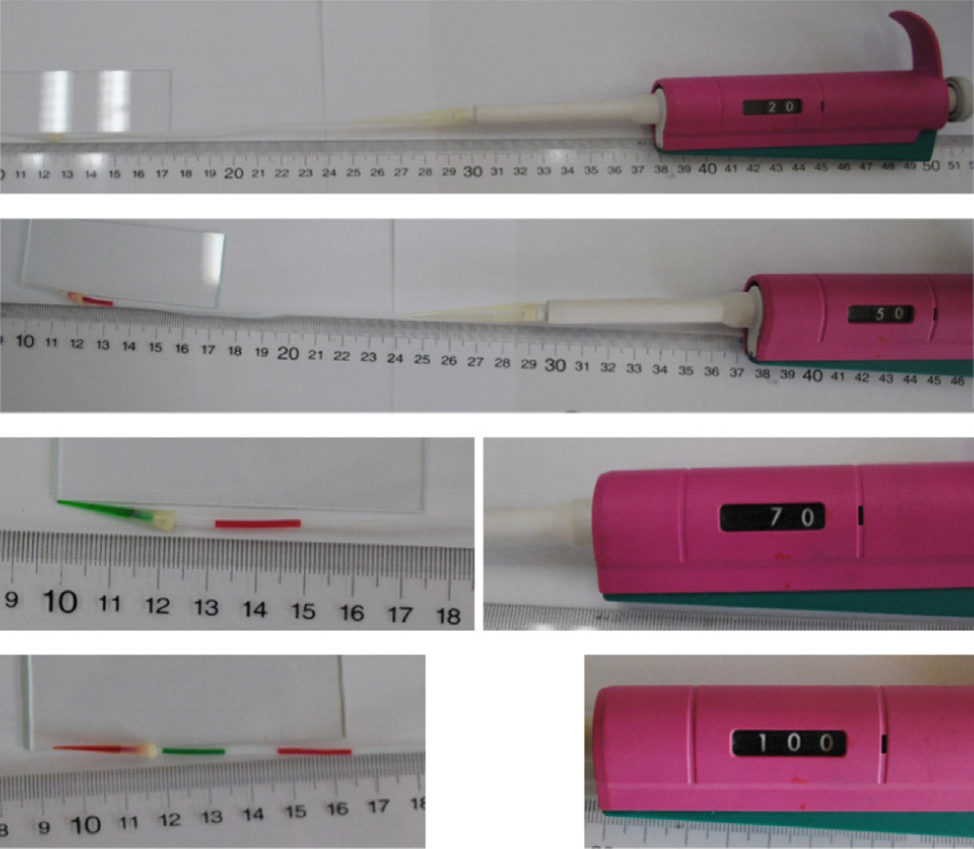
Reagents were loaded into Teflon tube with the help of pipettor.

For the DNA isolation form tissue, we introduced 70% (v/v) ethanol, 70% (v/v) ethanol, buffer MP3, and lysate orderly by adjusting the knob of pipettor. As shown in Fig. 2, the volume of these reagents and the space of two reagent segments could be controlled by the range that we adjusted the knob of pipettor.

**Fig. 3:**
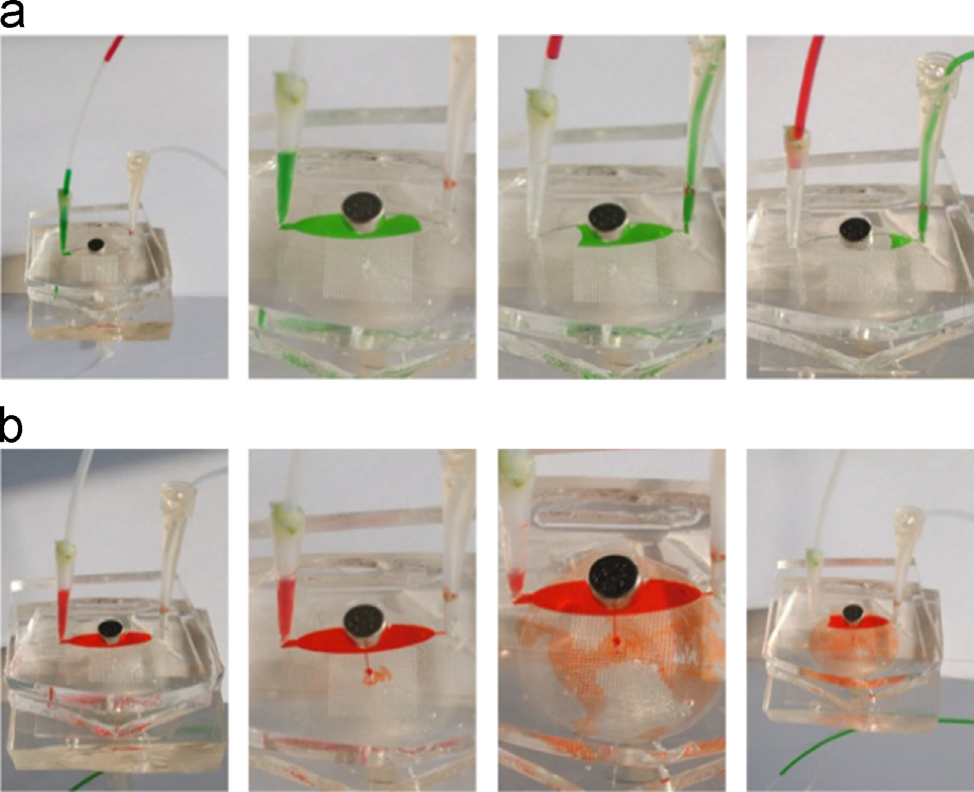
Schematic diagram of the process for flow control. (a) Sample waste and washing buffer (green) were sucked in the syringe avoiding contaminating the dPCR part by outlet 2. (b) PCR mix (red) eluted the DNA on magnetic particles and took them into the chambers under the negative pressure of μfilter.

Fluidic isolation of the NA extraction region and dPCR region was essential for the purposes of avoiding the incompatibility of the NA extraction with the amplification process. Because of the pull force from the negative pressure from the other side of the capture zone, the reagents could not seep into the channel that connected the dPCR module and NA extraction module, in case the reagents disturbed the subsequent dPCR reaction. As shown in Fig. 3, reagents solution were introduced into the NA isolation area from the Teflon tube by the syringe. The Mag-Bind particles were collected in the NA isolation area by the magnet, and the waste liquid was disposed into the syringe but the reagents did not seep into outlet 2. The μfilter provided negative pressure to sample loading and a syringe was used to pull the reagents for NA isolation. Then PCR mix was introduced into the NA isolation area from the Teflon tube, and took the DNA on the Mag-Bind particles into the dPCR part.

### 1.2 DNA extraction

The purification of NA from specimens was laborious and time-consuming until BOOM et al*.* [4] developed a rapid and simple method. Compared to conventional methods, the microdevices have shown advantages in low reagent and sample consumption, enhanced sensitivity, increased speed, etc. [5–7].

When 20 μL lysed sample with the Mag-Bind particles was pulled into the chip and passed through the capture zone by the negative pressure from other side, the Mag-Bind particles with NAs were collected by a magnet. Then the three sections of reagent in Teflon tube were pulled into the capture zone and washed away proteins and/or other contaminants on the Mag-Bind particles. Finally, DNA was eluted by PCR mix and introduced into the dPCR region for subsequent dPCR analysis.

**Fig. 4:**
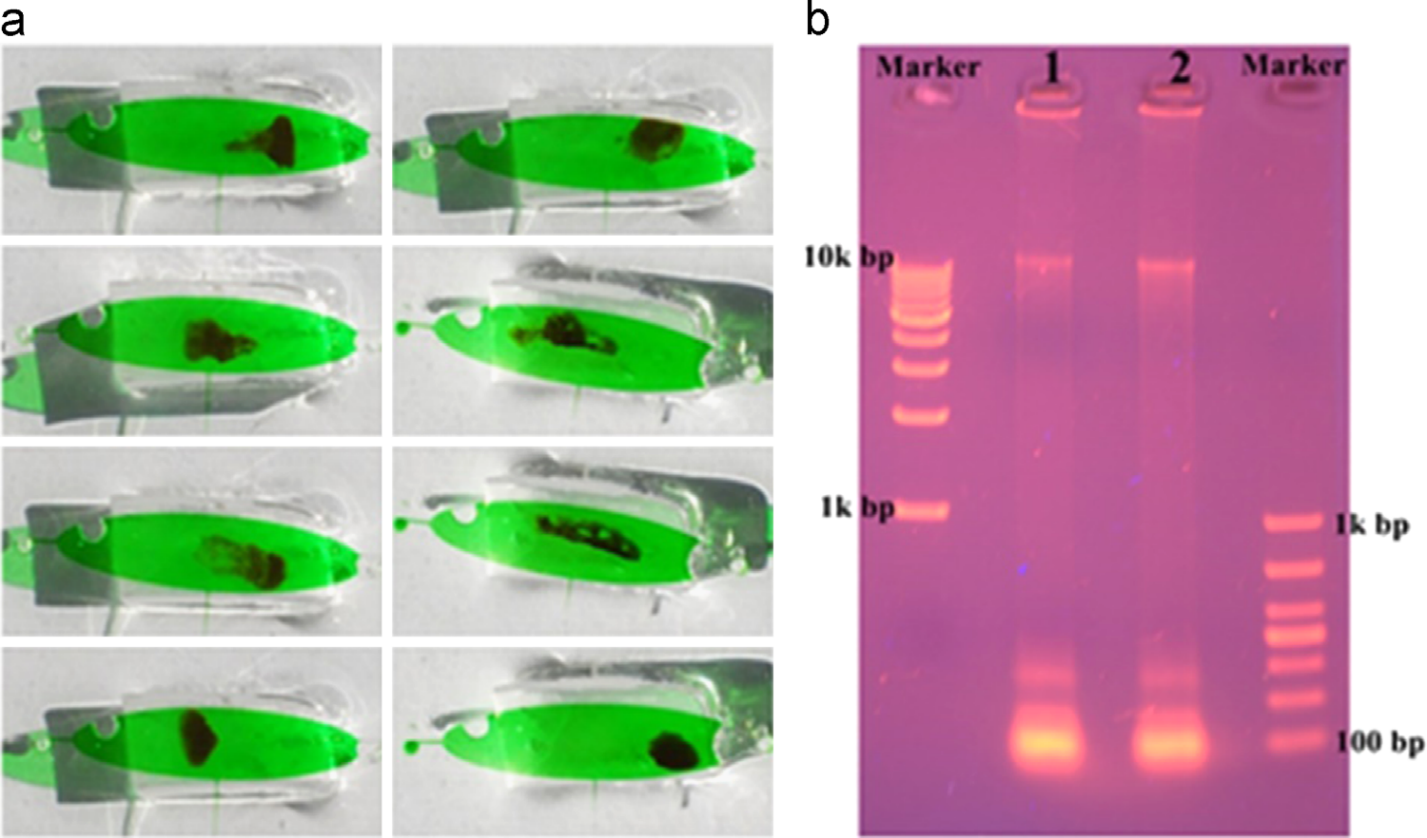
The elution efficiency of PCR mix. (a) The magnetic particles (black) in the capture zone were driven by the magnetic for paddling the reagent. (b) Genomic DNA on magnetic particles was eluted by PCR mix. Lane 1 was the PCR result of the Genomic DNA from 0.2 mL tube, and lane 2 was the PCR result of the Genomic DNA from microdevice (the PCR result was 89 bp).

**Fig. 5:**
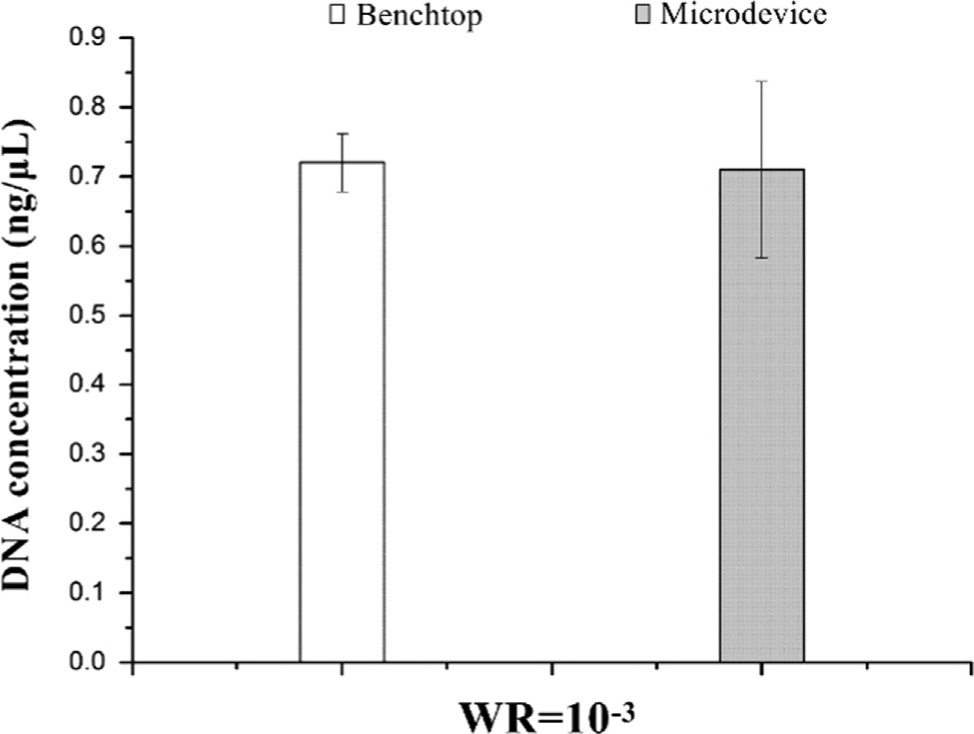
Average concentrations obtained from two gradients with the microdevice compared to the benchtop method. Microdevice and benchtop experiments were performed in triplicate. Error bars depict 95% CI.

**Fig. 6:**
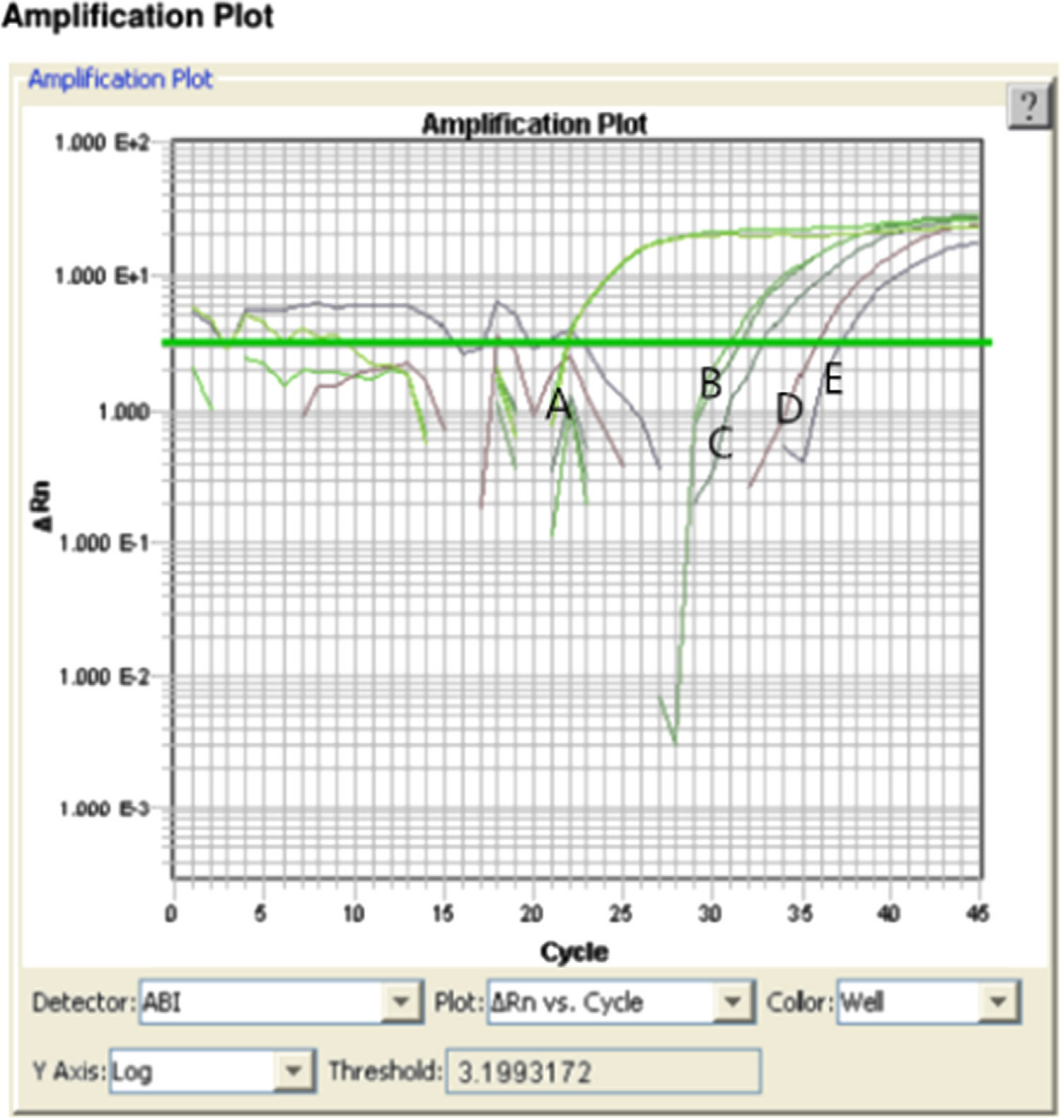
Amplification plot of different WR. A, WR=10^−3^; B, WR=10^−7^; C, WR=10^−8^; D, WR=10^−9^; E, WR=10^−10^.

But when the Mag-Bind particles were collected together and washed by 70% (v/v) ethanol, the Mag-Bind particles were clumped together and resisted washing and elution. We introduced a groove for magnet sliding above the NA capture zone to drive the magnetic particles movement around and improve the efficiency of washing and elution. The elution efficiency of PCR mix in a 0.2 mL tube and microdevice was determined by gel electrophoresis (Fig. 4). The genomic DNA and PCR results emerged in the same lane. The flow rates for wash reagents was 10 μL/min. Purified NAs obtained from benchtop and microdevice were quantified using real-time PCR with the Real Time PCR Bovine and Ovine DNA Detection Kit (TAKARA, RR913). Differences on quantified NA between benchtop and microdevice results were evaluated using the Mann–Witney *U* statistical test (SPSS 19.0, *P=*0.602>0.05) (Table 1). The data showed that there was non-significant differences between on-chip isolation compared to the benchtop procedure in DNA yields (Fig. 5). The bovine meat in different mixture with the ovine meat were detected by qPCR (Fig. 6).

## Conflict of interest statement

The authors declare that they have no competing financial interests.

## Figures and Tables

**Table 1:** The statistical analysis result of the real-time PCR.

Gradients	Samples	DNA concentration (ng/μL)	Average	Standard deviation	Results of Mann–Witney *U* statistical test
WR=10^−^^3^	1	0.7351	0.72	0.0423	0.602>0.05
1	0.6745
1	0.7491
1	0.7621
1	0.6726
			
2	0.8374	0.71	0.127
2	0.8599
2	0.6016
2	0.6384
2	0.617
