# Supplementary material for: Structure and fabrication details of an integrated modularized microfluidic system
Source: Data Brief. 2015 Oct 8;5:461–7. doi: 10.1016/j.dib.2015.09.036 (PMC4610953; doi:10.1016/j.dib.2015.09.036)
Supplement: Supplementary file 1 — Supplementary material [file mmc1.zip › Supplementary Table 1.docx]

**Table 1.** **The statistical analysis result of the real time PCR**

| Gradients | Samples | DNA concentration (ng/μL) | Average | Standard deviation | Results of Mann-Witney U statistical test |
| --- | --- | --- | --- | --- | --- |
| WR=10^-3^ | 1 | 0.7351 | 0.72 | 0.0423 | 0.602>0.05 |
|  | 1 | 0.6745 |  |  |  |
|  | 1 | 0.7491 |  |  |  |
|  | 1 | 0.7621 |  |  |  |
|  | 1 | 0.6726 |  |  |  |
|  |  |  |  |  |  |
|  | 2 | 0.8374 | 0.71 | 0.127 |  |
|  | 2 | 0.8599 |  |  |  |
|  | 2 | 0.6016 |  |  |  |
|  | 2 | 0.6384 |  |  |  |
|  | 2 | 0.617 |  |  |  |
