# Supplementary figures and images for: Structure and fabrication details of an integrated modularized microfluidic system
Source: Data Brief. 2015 Oct 8;5:461–7. doi: 10.1016/j.dib.2015.09.036 (PMC4610953; doi:10.1016/j.dib.2015.09.036)

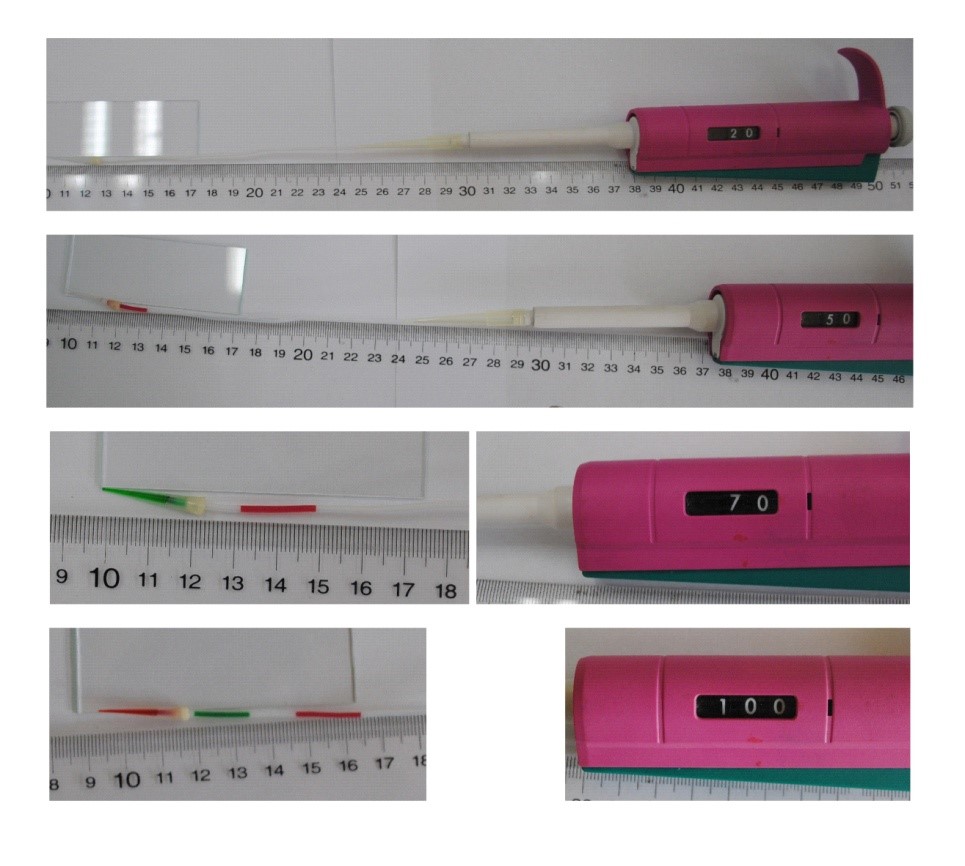

Supplement: Supplementary file 1 — Supplementary material [file mmc1.zip › Supplementary Figure 2.jpg]

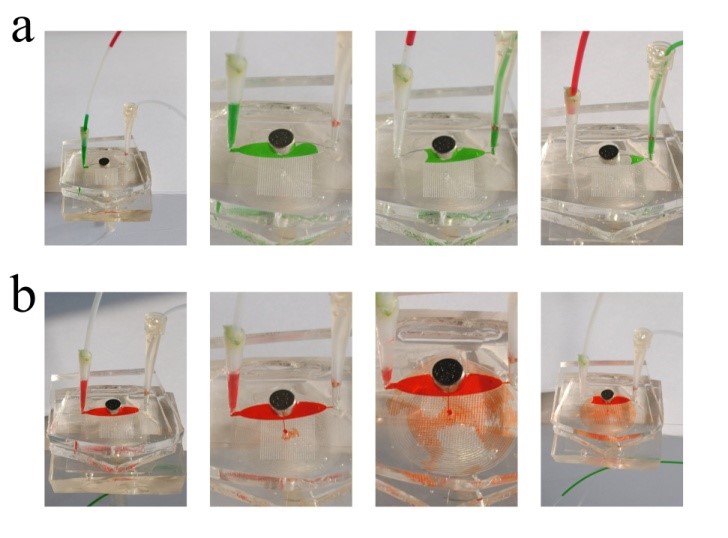

Supplement: Supplementary file 1 — Supplementary material [file mmc1.zip › Supplementary Figure 3.jpg]

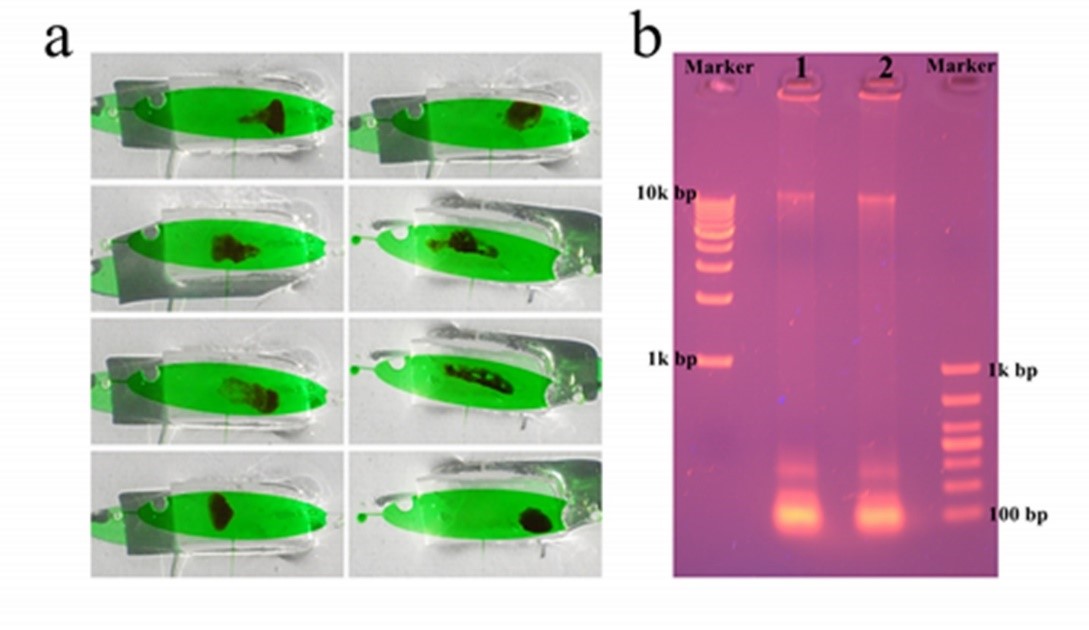

Supplement: Supplementary file 1 — Supplementary material [file mmc1.zip › Supplementary Figure 4.jpg]

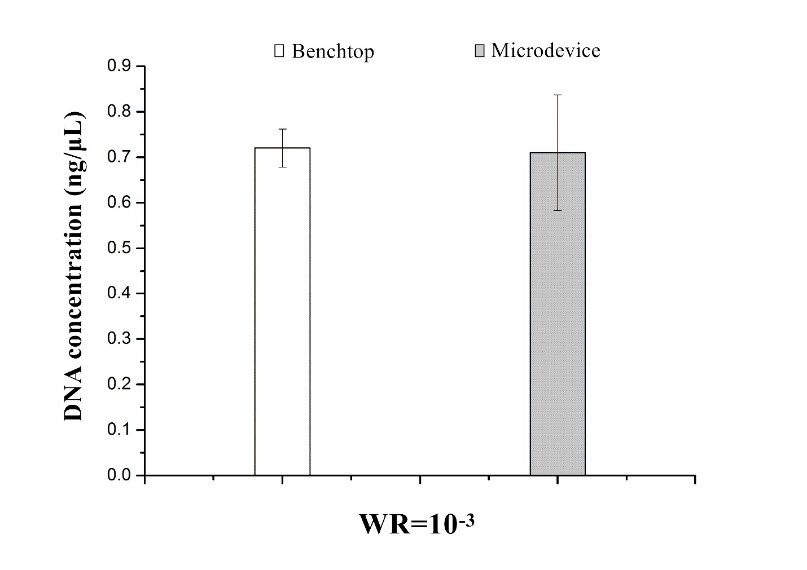

Supplement: Supplementary file 1 — Supplementary material [file mmc1.zip › Supplementary Figure 5.jpg]

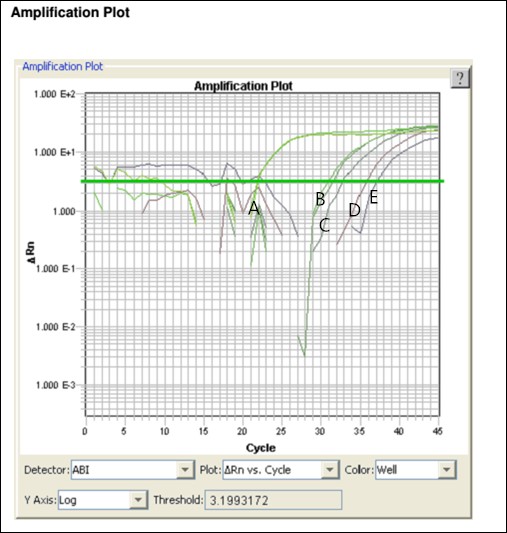

Supplement: Supplementary file 1 — Supplementary material [file mmc1.zip › Supplementary Figure 6.jpg]

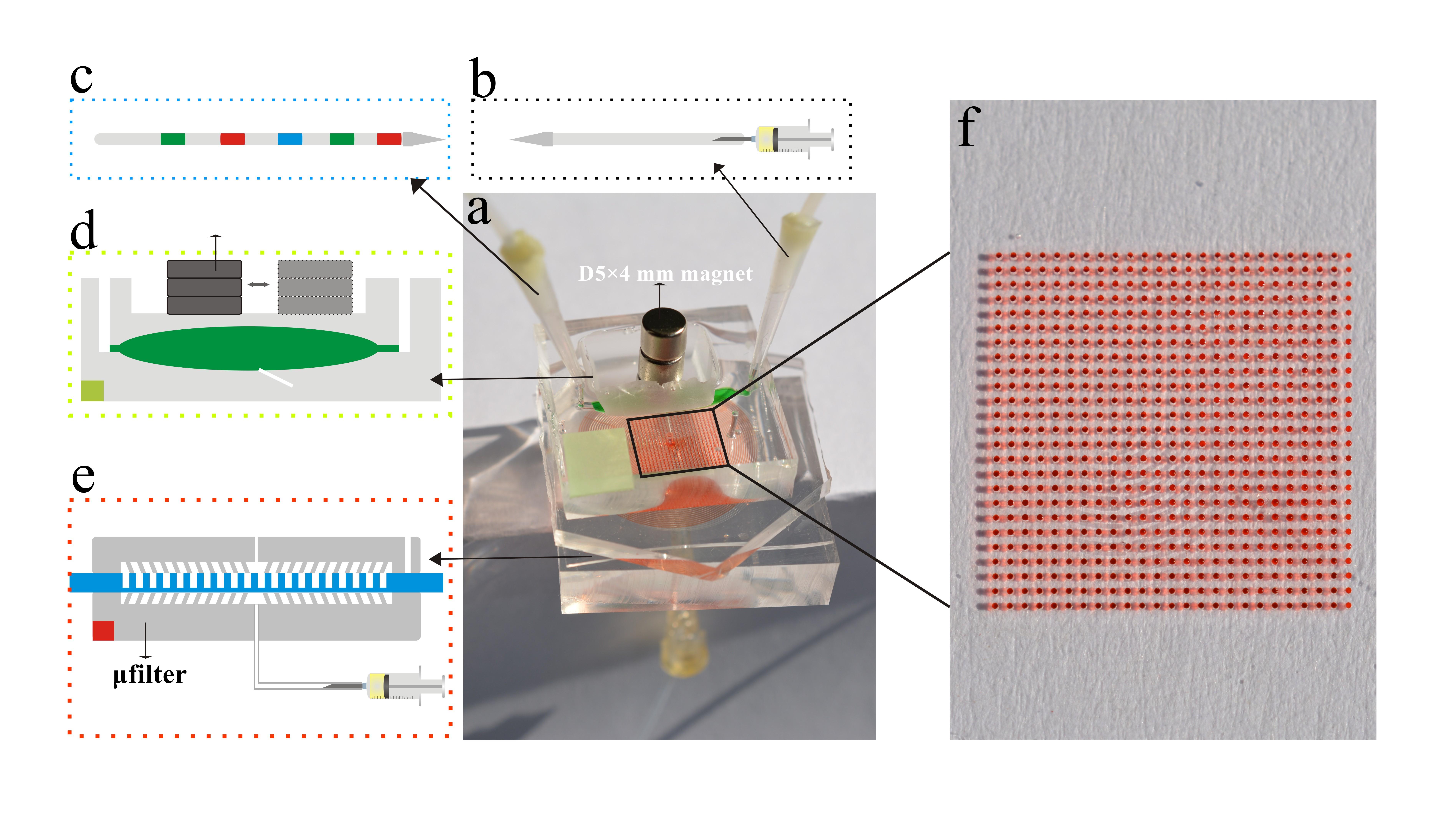

Supplement: Supplementary file 1 — Supplementary material [file mmc1.zip › Supplementary Figure 1.jpg]
