# Supplementary material for: Structure and fabrication details of an integrated modularized microfluidic system
Source: Data Brief. 2015 Oct 8;5:461–7. doi: 10.1016/j.dib.2015.09.036 (PMC4610953; doi:10.1016/j.dib.2015.09.036)
Supplement: Supplementary file 2 — Supplementary material [file mmc2.docx]

Competing interest statement：

The authors declare that they have no competing financial interests.
